# Inappropriate prescribing defined by STOPP and START criteria and its association with adverse drug events among hospitalized older patients: A multicentre, prospective study

**DOI:** 10.1371/journal.pone.0219898

**Published:** 2019-07-26

**Authors:** Mathumalar Loganathan Fahrni, Mohd Taufiq Azmy, Ezlina Usir, Noorizan Abd Aziz, Yahaya Hassan

**Affiliations:** 1 Department of Clinical Pharmacy and Pharmacy Practice, Faculty of Pharmacy, Universiti Teknologi MARA (UiTM), Selangor Branch, Puncak Alam Campus, Selangor Darul Ehsan, Malaysia; 2 Collaborative Drug Discovery Research (CDDR) Group, Communities of Research (Pharmaceutical and Life Sciences), Universiti Teknologi MARA (UiTM), Selangor Darul Ehsan, Malaysia; 3 Management and Science University (MSU), Off Persiaran Olahraga, Selangor, Malaysia; University of Sydney, AUSTRALIA

## Abstract

**Objectives:**

To provide baseline information on inappropriate prescribing (IP), and to evaluate whether potentially inappropriate medications (PIMs), as defined by STOPP (Screening Tool of Older Persons' potentially inappropriate Prescriptions) criteria, were associated with preventable adverse drug events (ADEs) and/or hospitalization.

**Methods:**

We prospectively studied older patients (n = 301) admitted to three urban, public-funded hospitals. We scrutinized their medical records and used STOPP-START (Screening Tool to Alert Prescribers to Right Treatment) criteria to determine PIM and potential prescribing omissions (PPO) respectively- together these constitute IP. Prescriptions with PIM(s) were subjected to a pharmacist medication review, aimed at detecting cases of ADE(s). The vetted cases were further assessed by an expert consensus panel to ascertain: i) causality between the ADE and hospitalization, using, the World Health Organization Uppsala Monitoring Centre criteria, and, ii) whether the ADEs were avoidable (using Hallas criteria). Finally, percentages of PIM-associated ADEs that were both preventable and linked to hospitalization were calculated.

**Results:**

IP prevalence was 58.5% (n = 176). A majority (49.5%, n = 150) had moderate to severe degree of comorbidities (Charlson Comorbidity Index score ≥ 3). Median age was 72 years. Median number of medications was 6 and 30.9% (n = 93) had ≥8 medications. PIM prevalence was 34.9% (117 PIMs, n = 105) and PPO 37.9% (191 PPOs, n = 114). Most PIMs and PPOs involved overuse of aspirin and underuse of both antiplatelets and statins respectively. With every increase in the number of medications prescribed, the likelihood of PIM occurrence increased by 20%, i.e.1.2 fold (OR 1.20, 95% CI: 1.1–1.3). Among the 105 patients with PIMs, 33 ADEs (n = 33); 31 ADEs (n = 31) considered “causal” or “contributory” to hospitalization; 27 ADEs (n = 27) deemed “avoidable” or “potentially avoidable”; and 25 PIM-associated ADEs, preventable, and that induced hospitalization (n = 25), were identified: these equated to prevalence of 31.4%, 29.5%, 25.7%, and 23.8% respectively. The most common ADEs were masked hypoglycemia and gastrointestinal bleed. With every additional PIM prescribed, the odds for ADE occurrence increased by 12 folds (OR 11.8, 95% CI 5.20–25.3).

**Conclusion:**

The majority of the older patients who were admitted to secondary care for acute illnesses were potentially exposed to IP. Approximately a quarter of the patients were prescribed with PIMs, which were plausibly linked with preventable ADEs that directly caused or contributed to hospitalization.

## Introduction

Prescribing for older people is a complex process. Older patients are often excluded from premarketing drug trials because of ethical considerations or age-related physiological changes that affect pharmacokinetics and pharmacodynamics of drugs,. Therefore, recommended doses for older patients are habitually drawn from extrapolated data of a young, healthy population, which may be inappropriate. [1–2] In addition to appropriate dosing, prescribers have an obligation to address the intricacies of prescribing for a group of patients with multiple comorbidities requiring multiple medications. [3]

Historically, polypharmacy has been viewed negatively due to its association with drug-drug and drug-disease interactions. Hoewever, there is increasing recognition that polypharmacy can, in fact, be appropriate, as evidence-based guidelines regularly advocate the use of more than one drug in the management of certain chronic illnesses such as hypertension. [4] On the contrary, under-prescribing can deny patients essential pharmacotherapy and this can, in turn, pose risks to patients’ safety and well-being. [5]

Interestingly, while inappropriate prescribing (IP) is possible with polypharmacy, not every individual with polypharmacy will have had an incidence of IP. IP occurs in any of the following circumstances: prescribing of medications that pose more harm than benefit, particularly when safer alternatives exist; prescribing of inappropriate dose or duration of drugs; presence of clinically significant drug-drug and drug-disease interactions; under-prescribing of potentially beneficial medications, and duplication of agents. [6–9]

Pharmacovigilance is crucial for detecting uncommon yet potentially hazardous adverse drug events (ADEs). An ADE is defined as any untoward medical occurrence that may present during treatment with medication but does not necessarily have a causal relationship with the treatment. [10] One inherent challenge in pharmacovigilance is that many case reports concern suspected ADE (specifically, adverse drug reactions), and these reactions are rarely specific for a single drug. In addition, a rechallenge is seldom ethically justified, and diagnostic tests are usually unavailable. Furthermore, few adverse reactions are “certain” or “unlikely”; many fall between these extremes (i.e. are possible or probable). In an attempt to resolve these challenges, many structured systems have been developed for a synchronized assessment of causality. [11] For example, the World Health Organization–Uppsala Monitoring Centre (WHO-UMC) system for standardized case causality assessment [12] has a set of explicitly defined causality categories. Its categories are “certain”, “probable”, “possible”, “unlikely”, “conditional”, or “unassessable”. For causality to be categorized as “certain”, a gastrointestinal bleeding episode, for example, must have a plausible time relationship with warfarin intake. These causality assessments are usually carried out to highlight individual case reports, identify areas of uncertainty and reduce disagreement between assessors. None of the available assessments, however, have been demonstrated to yield a precise and reliable quantitative estimation of relationship likelihood. [13]

ADEs that stem from a fault in prescribing due to an erroneous medical decision or because of lack of knowledge or incomplete patient information or training can result in patient harm, and are in fact avoidable or preventable. In ambulatory, care home, and critical care settings, where the prevalence of chronic illnesses, dependency, and disabilities are high, exposure to inappropriate medications correlated with an increasing number of ADEs: these patients had higher incidences of adverse drug reactions, hospitalization rates, and mortality [13–15]. These, in turn, can have a significant impact on healthcare resources, public health, and costs utilization [2,3,8,11].

Measures of IP can be classified as explicit (criterion-based) or implicit (judgment-based). [16] Explicit criteria, for example, Beers criteria, Inappropriate prescribing for Elderly Tool (IPET), and STOPP (Screening Tool of Older Persons' potentially inappropriate Prescriptions) and START (Screening Tools to Alert Prescribers to Right Treatment)—were developed with the intention of providing tools for assessing the quality of prescribing in older people. [17] They show lists of drugs that could pose a high risk of ADEs for the elderly and were developed by consensus panels of healthcare professionals. They comprise sets of optimal prescribing indicators designed as a universal minimum standard that could be applied to all patients and can, therefore, be computerized and applied easily to a large database. [18] In contrast, implicit measures—for instance, Medication Appropriateness Index—[19] are highly patient-specific and therefore require not only access to a broad array of clinical data, but also the judgment of trained clinicians.

Using principally the Beers and START-STOPP criteria to assess patients’ prescriptions, the prevalence of potentially inappropriate medication (PIM) prescribed were reportedly 12% to 80% and were widespread among older adults living within the community, and at nursing homes and health institutions [17, 20] Findings of a systematic review of PIM prevalence in nursing homes revealed higher (49%, 95% CI 43–56) point prevalence estimates in European countries than those reported in North America (27%, 95% CI 17–37) or in other countries (30%, 95% CI 19–40). [21] Difference in prevalence rates were often attributed to the inclusion of varying age groups, availability of drugs, distinct data collection methods, and diversity in drug policy and drug marketing between countries. [22, 23]

Ultimately, improvements in prescribing and monitoring practices must translate into positive patient outcomes and reduce negative outcomes.[24] Although impacts of IP and ADEs on outcomes, such as hospitalization have been independently established, research demonstrating the causality and associations between the two is still in its infancy. [20, 25] Using a sample of older people living in the region of South East Asia, our study aimed to determine whether STOPP criteria PIMs were associated with ADEs, which resulted in hospitalization and, which were preventable. The objectives were to evaluate: (i) prevalence of IP, PIMs, potential prescribing omissions, PPOs, and ADE; (ii) relationships between patients’ demographic and clinical variables with PIM, PPO and ADE; (iii) degree of causality between the ADE and hospitalization; (iv) whether ADEs, which were causal and contributory to hospitalization, were avoidable, and (iv) percentages of patients with PIMs who had ADEs that were causal (and contributory) to hospitalization while also being avoidable.

The term ADE is frequently used interchangeably in literature with adverse drug reaction and occasionally with medication-related injury. An adverse drug reaction is viewed as the most common and observable or measurable part of ADE. The second type of ADE is an adverse drug withdrawal event characterized as clinical symptoms, which are related to the discontinuation of a drug. Lastly, the third type is therapeutic failure whereby the goals of treatment are not accomplished as a result of inadequate drug therapy. For consistency and to allow comparison with previous studies [25], the term ADE will be used throughout this article.

## Methods

### Study design and sampling

The study was prospective and conducted at three publicly-funded hospitals in cities within an hour’s drive of Malaysia’s capital city of Kuala Lumpur. Hospitals 1 (H1) and 2 (H2) had 620 beds each, and hospital 3 (H3) had 960 beds. The three hospitals are governed by the Ministry of Health and therefore, implementation of patient safety practices and prescribing policies were similar in each case.

There was no similar data for secondary care available for comparison, and accordingly we used PIM prevalence of 23.7% (i.e. expected prevalence or proportion, P = 0.237) which was derived from a study of nursing homes. [26] By using a sample size calculation software developed by Naing et al [27], the minimum sample size required for the study was 278.

We prospectively studied 301 consecutive patients admitted with acute illnesses to the three teaching hospitals (H1, n = 86; H2, n = 86; H3, n = 129) over a 7-month interval (June-December 2014). Patients were aged 65 years or older and were admitted via the Emergency Department and under the care of the general medical or surgical services as required. They were recruited based on the study’s inclusion (prescribed with at least 1 medication) and exclusion criteria (patients who were in a coma, admitted for elective procedures, or who had missing information in their medical records).

A research clinical pharmacist (M.T.A.) collected the data. In 90% of the cases, data were collected within the first 24 hours of admission before patients were transferred to various wards for admission. Details of patients’ demographic characteristics, current and previous medical diagnoses, history of substance use, comorbidity burden (Charlson Comorbidity Index score) [28], current regular prescription medicines, blood pressure values, hematological and biochemical profiles, and history of drug allergy or intolerance were all recorded. Nonprescription medication [over-the-counter (OTC) products and health supplements] intake was also recorded for the sake of completeness. These medications were not accounted for in the total number of medications taken, and were excluded from the analysis not having been considered an important cause of ADEs except in cases of overdose. However, they were addressed with prescribers in instances where a pharmaceutical care issue arose. Pharmaceutical care issues are problems in the pharmacotherapy of the individual patient that actually or potentially interfere with desired health outcomes, which include adverse drug reactions, drug choice problems, dosing problems, drug-use problem, and interactions.

Details of difficulties with activities of daily living (ADL) [29] were obtained from medical records as well as from patient and caregiver interviews. Patients who required assistance with performing day-to-day activities such as bathing, movement, dressing, going to the toilet, and feeding were categorized as “dependent”; patients who were able to perform two or more of these activities were categorized as “semi-dependent”, and patients who could perform all of the activities without limitations were categorized as “independent”. Information on ADL statuses was required for the application of one START criterion related to the cardiovascular system.

Ethical approval was obtained from the National Medical Research Registry (NMRR), Institute for Health Behavioral Research (IHBR) and Medical Research Ethics Committee (MREC) of the Ministry of Health [NMRR-13-1249-16821(IIR)]. Waiver of consent to review medical records was granted. Patients were anonymized and all identifiable personal information was removed.

### Measures

#### Phase 1: Screening with STOPP and START criteria

The 2008 STOPP- START criteria from Ireland [30] were utilized in this study to detect IP. STOPP consists of 65 indicators for potentially inappropriate medications (PIM), which commonly cause drug-drug and disease interactions, unnecessary therapeutic duplication, and which increased risks of cognitive decline and falls in older people. START consists of 22 indicators for medications that should be considered for a list of conditions provided no contraindication to prescription exists (prescribing omission). The criteria are arranged according to the physiological group e.g. cardiovascular, respiratory, etc and have demonstrated good validity, interrater-reliability, and applicability in the UK and Europe [31]. The updated version of the criteria—STOPP-START version 2 [32]—was not made available when the research group made submissions for ethical approval, and thus we continued to utilize the older version.

Detection of PIM and PPO was based on patients' medications at the point of hospital admission, prior to any modifications made by the admission team. STOPP-START criteria were applied to the combined patient clinical profile and juxtaposed medication list, and instances of PIM and PPO were recorded. The application of STOPP-START criteria was performed by a research clinical pharmacist (M.T.A). For a pilot sample of 20, an academic pharmacist (M.L.F) also performed the assessment and counterchecked the data. Both have expertise in geriatric pharmacotherapy and medication safety. Any differences were subsequently resolved through discussion. A previous study, in its inter-rater reliability analysis, using the κ statistic, found strong agreement between clinical and academic pharmacists when using STOPP [0.89 (CI 0.69‐1.0)] and START criteria [0.91 (0.75‐1.0)]. [33]

#### Phase 2: Medication review and assessment using World Health Organization–Uppsala Monitoring Centre (WHO- UMC) criteria

For the patients with at least one PIM in their prescriptions, the research clinical pharmacist scrutinized their medical records and performed a medication review. The review was aimed at identifying ADEs, which were ADRs or medication-related injuries (e.g. hypotension in a patient taking diuretics). We had not aimed to identify ADE related to underuse (therapeutic failure) or withdrawal of drugs, although information on these, if any existed, was recorded and relayed to the physician-in-charge of each patient. Admissions for intentional self-harm and drug abuse were also excluded. Medication reconciliation was done on-opportunities basis. The ADEs included in the analysis were those which occurred before hospital management began, i.e. ADEs pertaining only to preadmission prescription medications were identified and not those occurring during the hospitalization. Nevertheless, any ADE identified (including drug omission in pre- and post-admission) during the scrutiny of medical records would have been raised with physicians in-charge for intervention.

Following the medication review, the research clinical pharmacist handed the vetted cases of ADEs to a panel of 5 experts in geriatric pharmacotherapy (2 geriatricians [N.A.M and I.I.A] and 3 clinical pharmacists [P.C.W, T. S.S, and P.J]) affiliated with the Ministry of Health. The panel utilized the WHO-UMC set of criteria to assess the causality for adverse reactions (broadly ADEs) raised during the pharmacist medication review. If the main diagnosis responsible for the admission was potentially related to the ADEs, then the admission to inpatient wards was accounted for as ADE-related, as proposed and validated by Stausberg et al. [34] To ensure a temporal relationship between the ADE and corresponding medication, the medication was postulated to be have been started within 30 days prior to the admission. The 30-day observation period was selected as adverse reactions often manifest shortly after the administration of drugs. [35]

In this assessment, the panelists individually analyzed all ADEs. They were not made aware of any PIMs, which were detected during Phase 1. This method relies on “expert judgment” as opposed to the use of algorithms or probabilistic methods. The WHO-UMC criteria broadly state that all points should be reasonably complied with: chronological relationship between administration of the drug and the occurrence of the reaction or event, differential screening for other drugs or diseases, confirmation of reaction by in vivo or in vitro tests, response to withdrawal (if applicable), rechallenge (if necessary, taking into account ethical considerations), and known information on similar events. Indeed, the application of the criteria involved a combined assessment, in view of the clinical-pharmacological aspects of the case history and the quality of documentation of the observation [36]. A consensus agreement of at least 3 of the 5 expert panel was required to determine if the ADE caused or contributed to the hospitalization.

#### Phase 3: Assessment using hallas criteria

Following the application of WHO-UMC criteria, cases of ADEs that were “causal” (principal cause of hospitalization) or “contributory” (clinically significant contributory factor to the hospital admission), i.e. those that fulfilled criterion “certain”, “probable” or “possible” were further analyzed by the same expert panel to evaluate whether each ADR (broadly ADE) was “definitely avoidable”, “possibly avoidable” or “not avoidable” [37].

The process was comprehensive and required the same expert panel to convene to discuss each case in detail. References such as the British National Formulary (BNF) and reputable pharmacology texts were utilized. [38, 39] ADEs that were “definitely” or “possibly” avoidable contributed to the analysis. Generally, these were ADEs, which involved organ dysfunction, homeostatic dysregulation, age-related changes in pharmacokinetics and pharmacodynamics, and known drug–drug interactions that predictably and adversely influenced an individual’s drug handling and response mechanisms.

### Data analysis

All statistical analyses were performed using SPSS version 21. We first described the distribution of demographic characteristics. Continuous variables were expressed as the mean ± standard deviation (SD). Categorical variables were expressed as the proportion in percentage (%). An independent t-test was used to examine the continuous variables, while contingency tables were used to evaluate the categorical variables. Frequencies of patients in who PIM, PPO, and ADE were detected were calculated. Analyses were two-sided and the statistical significance level was set at α = 0.05 with a 95% confidence interval; a p-value ≤0.05 was considered as statistically significant. We then employed univariate and multivariate logistic regressions to estimate the odds ratio (OR) of potential risk factors of PIM, PPO, and ADE. Binary logistic regression analysis using the backward stepwise likelihood-ratio method was conducted. Age, sex, gender, ADL statuses, history of hospitalization, smoking statuses and alcohol intake, number of medications, number of comorbidities, CCI scores, and other variables with a p-value less than 0.10 in the univariate models were fitted into multivariate models, and a final model was selected by removing all insignificant variables. All crude and adjusted ORs were expressed. Correlation and Hosmer-Lemeshow Goodness-of-Fit Tests were done to select the best prediction model.

## Results

### Association between inappropriate prescribing, demographic predictors and hospitalization

The research clinical pharmacist required an average of 45–50 minutes per patient to evaluate medical records, in addition to examining patients’ admission and reconciliation notes. Data were prospectively collected for a total of 301 consecutive older patients, of which the majority were males, n = 165 (54.8%). The median age was 72 years (range, 65–84; interquartile range [IQR], 67–77 years). Nearly half of the patients (49.5%) had a Charlson Comorbidity Index score of 3 or higher, i.e. moderate to severe illnesses with a 1-year mortality rate of 52% (Table 1). A total of 1876 medications were prescribed for the 301 patients (median = 6 medications per patient) [range, 1–27; IQR, 5–10 medications per patient]. Twenty percent (20%) of patients were prescribed with 2–3 medications, 25% with 4–5, 25% with 6–7 medications and 31% with 8 and more medications (Table 1). Based on the BNF’s system of classification, cardiovascular drugs were the most commonly prescribed drugs (1090) followed by the endocrine group (277), nutrition and blood groups of drugs (141) and respiratory drugs (103). Patients were admitted to the medical, surgical and orthopedic wards.

**Table 1:** Association between demographic variables and i) PIM, ii) PPO and iii) ADE.

Demographic variables	Subjects (n = 301)					
	At least 1 PIM		At least 1 PPO		At least 1 STOPP’s PIM	
	Frequency	^a^ P-value	Frequency	^a^ P- value	Frequency	^a^ P- value
**Age**						
65–69	35	0.393	45	0.539	7	0.323
70–74	33		25		11	
75–79	21		28		8	
≥ 80	16		16		5	
**Sex**						
Male	61	0.403	56	0.121	19	0.444
Female	44		58		12	
**History of Hospitalization**						
No	12	0.937	14	0.783	5	^b^0.381
Yes	93		100		26	
**Substance use**						
Non-Smoker	83	0.937	100	*0.006	24	0.895
Smoker	5		1		1	
Ex-smoker	17		13		6	
Non-alcohol user	100	0.454	113	*0.045	30	^b^1.000
Alcohol user	5		1		1	
**ADL** Independent	79	0.132	84	0.597	27	
Independent	79	0.132	84	0.597	27	^b^ 0.293
Semi-Dependent	9		13		2	
Dependent	17		17		2	
**Place of stay**						
Home (community dwellers)	101	0.730	110	0.888	30	^b^1.000
Institution	4		4		1	
**Number of** **comorbidities**						
2–3	52	0.596	53	0.113	1	^b^0.919
4–5	34		41		16	
≥ 6	15		19		10	
**Number of** **medications**						
2–3	12	*0.004	18	0.579	5	0.56
4–5	20		31		6	
6–7	32		30		7	
≥ 8	41		35		13	
**Charlson Comorbidity Index**						
1–2	51	0.324	46	*0.0004	17	^b^0.971
3–4	39		57		11	
5–6	13		9		3	
≥ 7	2		2		0	
**At least 1 ADE causal or contributory to hospitalization**						
Without					8	*0.0001
With					25	

^a^ Chi-square test for goodness of fit

^b^ Fisher’s Test

*statistically significant at p≤0.05

PIM: potentially inappropriate medications

The research clinical pharmacist required an average of 15–17 minutes per patient to systematically apply the STOPP-START criteria. The prevalence of overall IP was 58.5% (n = 176), i.e. those with at least one STOPP’s PIM or at least one START’s PPO. One hundred and seventeen instances (117) of PIM according to STOPP criteria were identified (Table 2). These were distributed amongst 34.9% (*n* = 105) of the study sample with a majority of them (n = 92) having 1 STOPP’s PIM each. One hundred and ninety-one instances (191) of PPO according to START criteria were identified (Table 2). These were distributed amongst 37.9% (n = 114) patients with a majority of them (n = 55) having 1 PPO.

**Table 2:** Number of potentially inappropriate medications identified.

STOPP Criteria	Instances
Aspirin with no history of coronary, cerebral or peripheral vascular symptoms or occlusive arterial event	24
Beta-blockers in those with diabetes mellitus and frequent hypoglycemic episodes i.e. 1 episode per month	17
Glibenclamide or chlorpropamide with type 2 diabetes mellitus	16
Aspirin with a past history of peptic ulcer disease without histamine H2 receptor antagonist or proton pump inhibitor	10
PPI for peptic ulcer disease at full therapeutic dosage for > 8 weeks	9
Loop diuretic for dependent ankle edema only ie no clinical signs of heart failure	6
Thiazide diuretic with a history of gout and aspirin, clopidogrel, dipyridamole or warfarin with a concurrent bleeding disorder	6
Calcium channel blockers with chronic constipation	6
Aspirin, clopidogrel, dipyridamole or warfarin with a concurrent bleeding disorder	6
Benzodiazepines	3
Any duplicate drug class prescription e.g. two concurrent opiates, NSAIDs, SSRIs, loop diuretics, ACE inhibitors	3
Loop diuretic as first-line monotherapy for hypertension	2
TCA with an opiate or calcium channel blocker	2
Long-term use of NSAID (>3 months) for relief of mild joint pain in osteoarthritis	2
Alpha-blockers in males with frequent incontinence i.e. one or more episodes of incontinence daily	2
Use of diltiazem or verapamil with NYHA Class III or IV heart failure	1
Prolonged use (>1 week) of first-generation antihistamines	1
First generation antihistamines in patients with falls	1
**TOTAL instances of PIM (Number of patients with PIM)**	117 (105)

PIM: potentially inappropriate medications; PPI: proton pump inhibitors; NSAID: nonsteroidal anti- inflammatory; *kPa*: kilopascal; COPD: chronic obstructive pulmonary disease; ACE: angiotensin converting enzyme; ARB: angiotensin receptor blockers; FEV: forced expiratory volume

When adjusted for gender, age, ADL, CCI, number of comorbidities, smoking statuses and alcohol intake, there is a 20% increase in the likelihood of receiving a PIM with higher numbers of medications prescribed—i.e the odds of receiving a PIM is 1.2 fold greater (OR 1.20, 95% CI: 1.10–1.32, p = 0.035). Similar regression analysis for PPO found no association between the likelihood of a PPO occurrence and demographic variables although a CCI score of 3–4, i.e. moderate degree of comorbid illness (p = 0.004), history of smoking (p = 0.006) and alcohol intake (p = 0.045) were associated with having a PPO in unadjusted models.

There was a statistically significant association (p = 0.0001) between having a STOPP’s PIM prescribed and having at least one ADE, which caused or contributed to hospitalization (Table 1). Details outlined in the subsections below.

### Nature of inappropriate prescribing

Table 2 depicts the most commonly prescribed PIMs according to STOPP criteria. The most frequently occurring criterion was ‘Aspirin with no history of coronary, cerebral or peripheral vascular symptoms or occlusive arterial event’ with 24 instances (18.6% of total PIM). This was followed by ‘Beta-blockers in those with diabetes mellitus and frequent hypoglycemic episodes—i.e. 1 episode per month’ where there were 17 instances (13.1% of total PIM); and ‘Glibenclamide or chlorpropamide with type 2 diabetes mellitus criteria’ with16 instances (12.4% of total PIM).

Table 3 demonstrates the most common PPOs according to the START criteria. The most frequently occurring criterion was ‘Antiplatelet therapy in diabetes mellitus if one or more co-existing major cardiovascular risk factor present’ with 61 instances (30.8% of total PPO). This was followed by ‘Statin therapy in diabetes mellitus if one or more co-existing major cardiovascular risk factor present’, where there were 44 instances (22.2% of total PPO); and ‘Metformin with type 2 diabetes with or without metabolic syndrome’, with 40 instances (20.2% of total PPO).

**Table 3:** Number of potential prescribing omissions identified.

START Criteria	Instances
Antiplatelet therapy in diabetes mellitus if one or more co-existing major cardiovascular risk factor present	61
Statin therapy in diabetes mellitus if one or more co-existing major cardiovascular risk factor present	44
Metformin with type 2 diabetes with or without metabolic syndrome	40
Statin therapy with a documented history of coronary, cerebral or peripheral vascular disease, where the patient’s functional status remains independent for activities of daily living and life expectancy > 5 years	7
Aspirin or clopidogrel with a documented history of atherosclerotic coronary, cerebral or peripheral vascular disease in patients with sinus rhythm	6
Angiotensin converting enzyme (ACE) inhibitor with chronic heart failure	6
ACE inhibitor following acute myocardial infarction	6
Home continuous oxygen with documented chronic type 1 respiratory failure (pO2 < 8 kPa, pCO2 < 6.5 kPa) or type 2 respiratory failure (pCO2 > 6.5 kPa)	6
Regular inhaled beta 2 agonist or anticholinergic agent for mild to moderate asthma or COPD	4
Calcium & vitamin D supplement in patients with known osteoporosis (radiological evidence or previous fragility fracture or acquired dorsal kyphosis)	3
ACE inhibitor or ARB in diabetes with nephropathy i.e. overt urinalysis proteinuria or microalbuminuria (> 30mg/ 24 hours) ± serum biochemical renal impairment	3
Regular inhaled corticosteroid for moderate-severe asthma or COPD where predicted FEV1<50%	2
Aspirin in the presence of chronic arterial fibrillation, where warfarin is contra-indicated, but not aspirin	2
Biphosphonates in patients taking maintenance oral corticosteroid therapy	1
**TOTAL instances of PPO (Number of patients with PPO)**	191 (114)

pO2: partial pressure of oxygen; pCO2: partial pressure of carbon dioxide; *kPa*: kilopascal; COPD: chronic obstructive pulmonary disease; ACE: angiotensin converting enzyme; ARB: angiotensin receptor blockers; FEV: forced expiratory volume

### Adverse drug events

A total of 33 ADEs were identified by the research clinical pharmacist in n = 33 out of the 105 patients (31.4%) screened—i.e. one ADE per patient, as is shown in Table 4. The panel of experts agreed that 31 of ADEs (n = 31, 29.5%) were to be considered causal or contributory to the hospitalization—i.e. in which most of the causal relations were noted as “probable” and “possible”. The remaining 2 ADEs were deemed “unlikely” to have caused or contributed to hospitalization after laboratory findings revealed acute exacerbation of existing conditions. Of the 31 ADEs considered causal or contributory to hospitalization, 27 were deemed avoidable or potentially avoidable (n = 27, 25.7%). When judged clinically, the 4 ADEs categorized as “unavoidable”, were deemed to be so because the benefits of the “inappropriate” drugs outweighed the risks posed—i.e. in those cases, experts considered the medications to be more beneficial than harmful. Of the remaining 27, 25 were ADE associated with STOPP’s PIMs (n = 25, 23.8%).

**Table 4:** Adverse drug events classified as “causal” or “contributory” to hospitalization, “possibly” or “definitely avoidable” and attributed to STOPP-listed PIMs.

Description of ADE	i) Number of ADEs that were causal or contributory to hospitalization, n [number of ADE identified but unlikely to have caused or contributed to hospitalization]	ii) Number of ADEs that were causal or contributory to hospitalization and were deemed definitely or possibly avoidable, n [number of ADE which caused or contributed to hospitalization but were unavoidable]	iii) Number of ADEs that were causal or contributory to hospitalization and that were deemed definitely or possibly avoidable and attributed to STOPP- listed PIM,n [number of ADE which caused or contributed to hospitalization and were avoidable but not attributed to STOPP-listed PIM]
Masked hypoglycemic symptoms	6	5 [1]	5
Upper GI bleeding	5	4 [1]	4
Falls	5	5	**5**
Episodes of hypoglycemia	4	4	**4**
Constipation	3	3	**3**
Urinary Incontinence	2 [1]	2	**2**
Exacerbation of gout	1	1	**1**
Cough	2	2	0 [2]
Hyponatremia	2	1 [1]	1
Acute kidney injury	1 [1]	0 [1]	0
**Total**	**31 [2]**	**27 [4]**	**25 [2]**

ADE: adverse drug event; PIM: potentially inappropriate medications; GI: gastrointestinal; NSAID: non-steroidal anti-inflammatory drug

### Association between IP and ADE

PIM demonstrated significant correlations of medium, positive strength with ADE (Rho = 0.479; p = 0.05). After adjusting for age, sex, comorbidity, activities of daily living, and number of medications, the odds of an ADE occurring, increased by 12 folds with the prescription of each PIM listed in the STOPP criteria (OR 11.8, 95% CI: 5.20–25.3, p = 0.018).

## Discussion

### Applicability of STOPP-START criteria

Our study was adequately powered with a 5% degree of precision to estimate the prevalence of PIM among older patients who presented with acute illnesses for admission to secondary care. We conclude from this that the overall IP prevalence of 58.5% was in agreement with published literature; previous studies using STOPP-START criteria demonstrated a prevalence of approximately 20% for general practice, 35% for hospital admissions, and about 80% in the case of nursing homes. [17,20, 40]

When performing the medication review, our research clinical pharmacist spent 45–50 minutes per patient (typically having had 6 medications [range 1–27]). In another study, a clinical pharmacist spent an average of 52 minutes on each patient’s records [41], while in a long-term care facility in Australia, a pharmacist required 3 hours. [42] In contrast, when using the STOPP-START tool, and taking into account that 31% of our patients had a high number of medications—i.e. 8 or more—the research clinical pharmacist reported that the application of the tool was fairly simple and time-effective (15–17 minutes per patient), and viewed it as a sustainable alternative as compared with the traditional medication review.

The 2008 STOPP criteria demonstrated good applicability and transferability to Asia. It has been used in this region in various settings: for example, in China’s largest nursing home [43], in nursing homes in Malaysia [26], and in hospitals in Taiwan [44] and India [45]. Using the STOPP- START tool, our study’s overall IP prevalence (at least one STOPP’s PIM or at least one START’s PPO) was reported as 58.5%, and with the specific prevalence of STOPP’s PIM recorded at 34.9%. Gallagher et al (2008) [40] demonstrated a very similar finding of 35% PIM prevalence and when the study was conducted among older patients admitted to 6 European hospitals, revealing a PIM prevalence of 51.3% was revealed [46]. The research team in Ireland in their article Hamilton et al (2011) [47] reported a STOPP PIM prevalence of 56.2%. The settings of these studies were comparable to those outlined in the current study—i.e. consecutive acute admissions to hospitals. In Taiwan, prescriptions of patients newly discharged were scrutinized and 36.2% of the study subjects presented with at least one PIM (in these patients, medications prescribed in the wards were accounted for). [44]

Using the START criteria, one or more appropriate medications were omitted in 34.9% of the patients—a lower prevalence than that found by Barry (2007), who demonstrated that in the cases of more than half (57.9%) of acutely-ill, newly hospitalized elderly patients, at least one appropriate medication was omitted from their list of regular prescription medications. [48] The authors reasoned that in 67% of their sample population who belonged to the older age group of 75 and above, physicians focused on palliation and therefore could have omitted drugs deemed unnecessary. Similarly, in the European hospitals’ study, the omission rate was 59.4% and the authors attributed the increasing degree of co-morbidity (CCI≥ 2) and more advanced age-range of ≥85 years as predictors. In Taiwan, prescriptions of patients discharged were scrutinized and 41.9% of the study subjects presented with at least one PPO (medications prescribed in the wards were included). [44] All the afore-mentioned studies varied in their design and population sampled e.g. community-dwelling, hospital, geriatric clinic, and long-term care patients. Nevertheless, the current study, together with those from other Asian countries, namely Taiwan [49], Thailand [50] and South Korea [51], concur with global practice and policy of avoiding the prescription of drugs when there is a lack of clear-cut evidence of therapeutic effectiveness in the older, aged population.

### Clinical relevance of inappropriate prescribing screening tools

There is potential for explicit criteria to be used as screening tools for not only IP but also as a method for identifying potential ADEs in older people. When compared with a similar tool, such as the Beers criteria, the STOPP criteria identified a higher quantity of medications associated with adverse drug events than the 2002 version of the Beers criteria [20], suggesting greater relevance as a clinical tool. [47] A newer study found that along with more instances of inappropriate prescribing, STOPP-START version 2 also targeted significantly more PIMs and PPOs associated with preventable admissions. [52] Similar comparison studies between the newer versions of Beers 2018 and STOPP-START version 2 are yet to be published as of March 2019.

Based on the current study, the STOPP-START criteria acted as a trigger tool for identifying IP and establishing a correlation to adverse health outcomes. In particular, when our STOPP-START audit prompted for caution with beta-blockers and antiplatelet use, this corresponded accurately with ADEs yielded—i.e. masked tachycardia in insulin-induced hypoglycemia as well as NSAID-induced gastrointestinal bleeding were identified. Extrapolating from our results, STOPP identified patients with a history of falls receiving potentially inappropriate psychoactive or vasodilator medications. In addition, in patients presenting with an isolated fall, STOPP identified several inappropriate prescriptions—e.g. long-acting benzodiazepines, first-generation antihistamines and inappropriately prescribed opiates. In parallel, the team of geriatricians and clinical pharmacists determined the causality and avoidability of many of the corresponding ADEs. Eventually, a high percentage of such ADEs were revealed as preventable and attributable to the drugs flagged as high-risk by STOPP-START which tends further to substantiate the clinical relevance of the criteria [53]. As for the WHO-UMC and Hallas criteria, the panel noted they entailed a short but significant learning curve, which may have led to higher application times until competence with them developed. The strategy adopted in our study was reliable in identifying potential ADEs, which often presented with non-specific symptoms such as confusion, falls with a resultant injury or constipation. Although falls are clearly multifactorial in nature, reviewing the medications of older adults is an essential component of comprehensive falls assessment. [54]

In our study, the two most evident omissions in patients’ prescriptions were: i) *antiplatelet therapy and ii) statin therapy*, *for diabetes mellitus if one or more co-existing major cardiovascular risk factor(s) present*. This was echoed in the research from Taiwan [44], where statin therapy followed by antiplatelet therapy for diabetes were most common. In Ireland [9], omissions of *statin therapy in diabetes mellitus and in symptomatic cardiovascular disease* were common occurrences. Hamilton (2011) echoed with similar findings where these were also the most common omissions. In their identical study, Barry et al (2007) [48] demonstrated that the top 3 PPOs were statins in symptomatic cardiovascular disease, warfarin in chronic atrial fibrillation and ACE inhibitor in chronic heart failure. In Belgium for instance, where a similar study was conducted, PIM involved overuse and/or misuse of benzodiazepines, aspirin, and opiates while omissions were mainly related to underuse of calcium and vitamin D supplementation, aspirin and statins. [15] The authors attributed hospitalization to PIMs in 27.1% of the patients (vs 29.5% in the current study).

Evidently, the nature of IP and ADE in our study denoted the common comorbidity burden faced in Asia–a region in which type 2 diabetes mellitus is highly prevalent in this region (Asia Pacific vs Europe). [55] As to the possibility that this pattern of antiplatelet and statin under-prescribing is due to the ageism phenomenon continues to remain unexplored. In STOPP-START Version 2 however, indicators for statin therapy and aspirin for primary prevention of cardiovascular diseases in diabetes mellitus (as they had been outlined in version 1) were removed because of weak or equivocal supporting evidence. [32]

At this juncture, we must reiterate that ADEs identified were ascribed to harm that occurred during normal care and usual doses prescribed for patients. Needless to say, prescribing guidelines and indicators for the “oldest” and “older” aged population is very much desirable.

### Predictor factors for IP and ADE

The median age of patients sampled in our study was 72 years (maximum age 84). The median ages of the population sampled in similar studies–i.e. at Taiwan’s veterans’ hospital [44] and at Ireland’s teaching hospitals [47] were 79 years and 77 years respectively.

Other baseline characteristics of the patients in our study were similar to that of Hamilton’s (2011). In both of the studies, there were equal proportions of males and females. While in the present study, 31% of the patients consumed ≥ 8 medications, 20% of those in Hamilton’s consumed ≥ 10 medications. In addition, 45% of the patients in this study consumed ≤ 5 medications daily while the corresponding figure in Hamilton’s was 34%. A greater proportion of patients in this study had severe illnesses (CCI ≥ 3) than those in Hamilton’s. The median number of medications per patient was 6 in both studies. A median of 7 medications per patient was typically evident in other studies; likewise, a high percentage of patients were prescribed with 8 and more daily medications [20, 56].

The likelihood of a PIM being prescribed increased with the prescription of greater numbers of medications. Polypharmacy and its close linkage with inappropriate prescribing have long been established. [57,58] This association can be explained by the presence of multi-morbidities in older adults, thereby necessitating multi medications. With additional comorbidities, additional exposure to a greater number of medications, as well as to new prescribers and specialists arise. With a greater number of medications, there are predisposing risks for errors or inappropriateness in prescribing in addition to duplication of medications and the prescribing of unnecessary medications. These in turn increase the chances for drug-drug interactions and adverse drug reactions to occur. Unrecognized adverse drug reactions can be treated as new disorders, being prescribed with further additional medications, a situation that leads to a phenomenon known as “prescribing cascade”. The explanation is supported clinically, where a study of older people revealed that having 5 drugs and greater, was a predictor factor for being prescribed with a PIM [44]. In a recent consensus initiative, developing guidelines and standard operating procedures for high-risk patients, medications, and contexts were concluded as top prioritization research areas. [59]

On the contrary, we noted that predictor factors such as higher CCI (lower expected survival time) were associated with increased probability of an omission occurring- possibly attributable to physicians focusing on palliation and improving the quality of life of patients. In our study, apart from having a CCI score of 3 and above, having a history of smoking and alcohol intake were associated with omission occurrence. One probable explanation could be that these individual factors–e.g. decreased renal function, and past exposure to substances such as alcohol and tobacco—collectively form a risk stratification and guiding decision to avoid prescribing, and hence exposure to drugs. Somewhat counter-intuitively, findings of one study suggested that patients taking more than five medications (43%) were more prone to underprescribing than those who were taking four medications or fewer (13.5%). [60] However, Steinman and colleagues argued that although underuse was common, the total number of medications did not affect it. [61]

### Strengths and limitations

The number of preventable ADEs (27 of 31) identified in 25.7% of patients screened and the number of ADEs attributed to clinically relevant PIM i.e. 25 of 27 (23.8% of patients screened could potentially indicate that the ADEs may have resulted from suboptimal care (i.e. an erroneous prescribing decision) as opposed to those that arose from appropriate care (non-preventable ADE). Nevertheless, one must be mindful that only a pool of prescriptions already comprising of PIMs was screened for ADE. The selection of cases with predefined outcomes of PIM and hospitalization was a limitation. However, given the prospective nature of the multicenter study, a timely medication review was crucial as intervention and assessment were to be done while patients were still hospitalized. In addition, further adjudication by the panel to determine temporal causality (Hallas criteria requires physician to intervene–e.g. to withhold a drug) was necessary. In addition, although hospital admission was accounted for as ADE-related if the main diagnosis responsible for the admission was potentially related to ADEs, under real-time conditions, detection of ADEs in older patients with multi-morbidities and taking several drugs is complicated.

Many studies to date have been retrospective, and have been conducted in the community or long term care-based institutions. The effects on patient-centered outcomes were less well-defined. Several systematic reviews have concluded that there was only weak evidence of an association between PIMs and adverse patient outcomes, and they have emphasized that further research is needed. [62] In our prospective study, we demonstrated a causative relationship between the process measure of prescribing and patient harm. Additionally, many studies had focused on inappropriate medication alone when determining IP. In contrast, our study adds to literature the rate of omission of appropriate, evidence-based medications.

Although a non-probability method is prone to sampling bias, enrollment of consecutive admissions to hospitals has the advantage of revealing a true unselected sampling or cross-section of the type of patients admitted, the nature and extent of their illnesses, the treatments applied as well as the progress observed. Moreover, as our study was prospective in nature, it began, and its ethical review took place, before the clinical outcomes of interest had occurred, and before any data were recorded. While no causality assessment method is universally accepted, expert judgment is the most widely used to evaluate the relationship between drug treatment and the occurrence of an adverse event.

Our sample may not be representative of the diverse elderly population living in the country. Nevertheless, the characteristics of our sample did not defer significantly from the 2017 health facts published by the nation’s Ministry of Health Informatics Centre, in which diseases of the respiratory and circulatory systems were attributed to the top 5 principal causes of hospital admissions.

Albeit only using one rater in our study, having two or more researchers apply the criteria on the same patients’ medical lists can increase internal consistency (homogeneity) in the outcome of the evaluation. In addition, while an updated, more extensive and relevant version has since been published, we used the original version of STOPP-START and this may have impacted the applicability of the research findings on current practice.

The application of the STOPP-START criteria requires clinical information such as diagnoses and laboratory parameters, and consequently our research was dependent on the accuracy of the medication reconciliation process across healthcare transitions made prior to admission. Indeed, systematically applying an extensive set of criteria was time-consuming. While ideally, the records and databases were enriched with clinically validated data, in reality, the healthcare system may not have facilitated accurate and timely medication reconciliation during care transitions, which made it challenging for the research clinical pharmacist to perform a reliable medication review and to apply the criteria effectively.

Our study focused only on inappropriate prescribing prior to hospital admissions. The practice of prescribing in primary care is mostly repeated prescribing of medications for chronic diseases that are often prescribed for patients initially in secondary care. This posed an additional challenge for the researcher in terms of complete medication reconciliation, particularly when multiple healthcare providers (at times from multiple institutions) attended the patients. Further investigations are required to give insights into the underlying reasons and justifications, if any, for prescribing or omitting medications listed in STOPP-START by the general practitioners or specialists who attended the patients.

### Implications to practice and future directions for research

Future work should explore the effect of interventions targeting IP on the detrimental consequences of IP on clinical, humanistic and economic outcomes. Exploration of factors underlying IP should undertake quantitative as well as qualitative methods, using more in-depth observational and longitudinal research methods.

Nationwide studies in both primary and secondary care settings, might include matched larger samples of older patients and assess whether adverse drug events were more likely in community dwellers or hospitalized older patients. Ideally, all patients sampled would be assessed for adverse events as opposed to only those whose prescriptions had been flagged as inappropriate. In order to underscore the credibility and clinical relevance of the START-STOPP tool at the individual patient level, each PIM identified should be reiterated by an expert’s input. For example, if the expected benefit of a particular medication was judged to outweigh the potential harm, such as an antipsychotic drug in a patient with schizophrenia, the PIM would be assessed by an expert as not clinically relevant, as in our study, which showed 4 instances in which ADE was contributed by “inappropriate” medications. This was according to the experts who designed STOPP-START, who had assessed the inappropriate medications as high-risk at the general population level. Yet, at an individualized patient level, such medications were of relevance, credible, and acceptable.

In Europe, for instance, clinically relevant PIM can be utilized for quality improvement initiatives and as indicators for training modules of critical and primary care trainees. There is a need to train current and future physicians to firstly recognize and then take remedial actions when faced with a prescribing cascade phenomenon as opposed to accurately diagnosing an exacerbation of a medical condition. Recommendations to reduce prescription errors and prescribing faults—i.e. wrong clinical decisions—include education and training of prescribers and the use of electronic aids such as computerized physician order entry with computerized clinical decision support systems to minimize such errors. [63] To evaluate such implementations, feedback control mechanisms need to be in place. This highlights the need for periodical audit and medication review incorporating situational analysis in which the pharmacist gathers, analyzes and interprets information about patient’s clinical conditions and pharmacotherapy, aiming to evaluate his or her drug-related needs. There is however a need for an international agreement on the medication review process. [42],

Equally, a reliable method of assessing the relationship between the administration of a drug and an adverse clinical event is warranted. Trigger tools such as the Drug-related admission Adjudication (DRA) guide to screen for drug-related hospital admissions in older persons that considers multiple co-morbidities and multiple medications in older people are urgently warranted. [64] It remains to be seen whether the widespread utilization of these tools reduces the incidence of ADEs and the associated morbidity and mortality. Moreover, to remain up-to-date clinically, the use of prescribing appropriateness tools or criteria must effectively translate into the optimization of prescribing practices—for example, consider changing evidence base, factoring pharmacovigilance of newly licensed, and cater for the ever-growing body of literature and allowing extension of criteria and eliciting reduction in rates of ADEs. Ultimately, an extrinsic method of assessment with good predictive validity, sensitivity and specificity would be desirable, allowing for extensive application in all settings pertinent to older people.

## Conclusion

The study provided baseline information on inappropriate prescribing and deduced plausible links between potentially inappropriate medications with both hospitalization and preventable adverse drug events. In sustaining primary and secondary disease prevention, it is crucial for physicians, pharmacists and healthcare professionals alike to adopt a multidisciplinary approach to ensure that older patients are prescribed and ultimately receive, appropriate and indicated medications. Failure to do so, as demonstrated in this study, could impact clinical outcomes, thus underscoring the importance of addressing these intricacies and delivering multifaceted patient-centered services to a vulnerable population.

## References

[1] Loganathan M, Singh S, Franklin BD, Bottle A, Majeed A. Interventions to optimise prescribing in care homes: systematic review. *Age and ageing*. 3 2011; 40(2):150–162. 10.1093/ageing/afq161 21262782

[2] Miller GE, Sarpong EM, Davidoff AJ, Yang EY, Brandt NJ, Fick DM. Determinants of Potentially Inappropriate Medication Use among Community-Dwelling Older Adults. *Health services research*. 2017; 52(4):1534–1549. 10.1111/1475-6773.12562 27686781 PMC5517671

[3] Boparai MK, Korc‐Grodzicki B. Prescribing for Older Adults. *Mount Sinai Journal of Medicine*. 2011; 78: 613–626. 10.1002/msj.20278 21748749

[4] Hughes CM, Cadogan CA, Patton D, Ryan CA. Pharmaceutical strategies towards optimising polypharmacy in older people. *International journal of pharmaceutics*. 2016; 512(2):360–365. 10.1016/j.ijpharm.2016.02.035 26921516

[5] Cadogan CA, Ryan C, Hughes CM. Appropriate Polypharmacy and Medicine Safety: When Many is not Too Many. *Drug safety*. 2016;39(2): 109–16. 10.1007/s40264-015-0378-5 26692396 PMC4735229

[6] Gallagher P, Barry P, O'Mahony D. Inappropriate prescribing in the elderly. *Journal of clinical pharmacy and therapeutics*. 2007; 32(2):113–121. 10.1111/j.1365-2710.2007.00793.x 17381661

[7] Beers MH. Explicit criteria for determining potentially inappropriate medication use by the elderly. An update. *Archives of internal medicine*. 1997; 157(14): 1531–1536. 9236554

[8] Wauters M, Elseviers M, Vaes B. Too many, too few, or too unsafe? Impact of inappropriate prescribing on mortality, and hospitalization in a cohort of community- dwelling oldest old. *British journal of clinical pharmacology*. 2016; 82(5):1382–1392. 10.1111/bcp.13055 27426227 PMC5061799

[9] Ryan C, O'Mahony D, Byrne S. Application of STOPP and START criteria: interrater reliability among pharmacists. *The Annals of pharmacotherapy*. 2009;43(7):1239–1244. 10.1345/aph.1M157 19584381

[10] The Importance of Pharmacovigilance—Safety Monitoring of Medicinal Products. WHO Collaborating Centre for International Drug Monitoring, Uppsala Monitoring Centre, 2002: 52 Available from http://apps.who.int/medicinedocs/en/d/Js4893e/#Js4893e.

[11] Meyboom RHB, Royer RJ. Causality Classification in Pharmacovigilance Centres in the European Community. *Pharmacoepidemiology and Drug Safety*. 1992; 1:87–97.

[12] The use of the WHO-UMC system for standardized case causality assessment. World Health Organization (WHO)—Uppsala Monitoring Centre. [Last accessed on 2019 Mar 16]. Available from: http://www.who-umc.org/Graphics/24734.pdf.

[13] Jano E, Aparasu RR. Healthcare outcomes associated with beers' criteria: a systematic review. *The Annals of pharmacotherapy*. 2007;41(3):438–447. 10.1345/aph.1H473 17311835

[14] Hiance-Delahaye A, de Schongor FM, Lechowski L. Potentially inappropriate prescription of antidepressants in old people: characteristics, associated factors, and impact on mortality. *International psychogeriatrics*. 2018; 30(5):715–726. 10.1017/S1041610217002290 29145919

[15] Dalleur O, Spinewine A, Henrard S, Losseau C, Speybroeck N, Boland B. Inappropriate Prescribing and Related Hospital Admissions in Frail Older Persons According to the STOPP and START Criteria. *Drugs & Aging*. 2012; 29(10):829–837. 23044639 10.1007/s40266-012-0016-1

[16] Kaufmann CP, Tremp R, Hersberger KE, Lampert ML. Inappropriate prescribing: a systematic overview of published assessment tools. *European Journal of Clinical Pharmacology*. 2014;70(1):1–11. 10.1007/s00228-013-1575-8 24019054

[17] García-Gollarte F, Baleriola-Júlvez J, Ferrero-López I, Cruz-Jentoft AJ. Inappropriate drug prescription at nursing home admission. *Journal of the American Medical Directors Association*. 2012;13(1):83–e9. 10.1016/j.jamda.2011.02.009 22208763

[18] Projovic I, Vukadinovic D, Milovanovic O, Jurisevic M, Pavlovic R, Jacovic S, Jankovic S, Stefanovic S. Risk factors for potentially inappropriate prescribing to older patients in primary care. *European Journal of Clinical Pharmacology*. 2016;72(1):93–107. 10.1007/s00228-015-1957-1 26416101

[19] Spinewine A, Schmader KE, Barber N, Hughes C, Lapane KL, Swine C, Hanlon JT. Appropriate prescribing in elderly people: how well can it be measured and optimised?. *The Lancet*. 2007;370(9582):173–84. 10.1016/S0140-6736(07)61091-5 17630041

[20] Hill-Taylor B, Sketris I, Hayden J, Byrne S, O'Sullivan D, Christie R. Application of the STOPP/START criteria: a systematic review of the prevalence of potentially inappropriate prescribing in older adults, and evidence of clinical, humanistic and economic impact. *Journal of Clinical Pharmacy and Therapeutics*. 2013;38(5):360–372. 10.1111/jcpt.12059 23550814

[21] Morin L, Laroche ML, Texier G, Johnell K. Prevalence of potentially inappropriate medication use in older adults living in nursing homes: a systematic review. *Journal of the American Medical Directors Association*. 2016;17(9):862–e1. 10.1016/j.jamda.2016.06.011 27473899

[22] Ghadimi H, Esmaily HM, Wahlstrom R. General practitioners' prescribing patterns for the elderly in a province of Iran. *Pharmacoepidemiology and drug saf*ety. 2011;20(5):482–7. 10.1002/pds.2106 21523850

[23] Bradley MC, Motterlini N, Padmanabhan S, Cahir C, Williams T, Fahey T, Hughes CM. Potentially inappropriate prescribing among older people in the United Kingdom. *BMC Geriatrics*. 2014;14(1):72. 24919523 10.1186/1471-2318-14-72 PMC4091750

[24] Lehnert T, Heider D, Leicht H, Heinrich S, Corrieri S, Luppa M, Riedel-Heller S, König HH. Health care utilization and costs of elderly persons with multiple chronic conditions. *Medical Care Research and Review*. 2011; 68(4):387–420. 10.1177/1077558711399580 21813576

[25] Hamilton HJ, Gallagher PF, O'Mahony D. Inappropriate prescribing and adverse drug events in older people. *BMC Geriatrics*. 2009; 9:5 10.1186/1471-2318-9-5 19175914 PMC2642820

[26] Chen LL, Tangiisuran B, Shafie AA, Hassali MA. Evaluation of potentially inappropriate medications among older residents of Malaysian nursing homes. *International Journal of Clinical Pharmacy*. 2012;34(4):596–603. 10.1007/s11096-012-9651-1 22622593

[27] Naing L, Winn T, Rusli BN. Practical issues in calculating the sample size for prevalence studies. Archives of Orofacial Sciences. 2006; 1: 9–14.

[28] Charlson ME, Pompei P, Ales KL, MacKenzie CR. A new method of classifying prognostic comorbidity in longitudinal studies: development and validation. *Journal of Chronic Diseases*. 1987; 40(5):373–383. 3558716 10.1016/0021-9681(87)90171-8

[29] Noelker LS, Browdie R. Sidney Katz MD: A new paradigm for chronic illness and long-term care. The Gerontologist. 2013;54(1):13–20. 10.1093/geront/gnt086 23969255

[30] Gallagher P, Ryan C, Byrne S, Kennedy J, O'Mahony D. STOPP (Screening Tool of Older Person's Prescriptions) and START (Screening Tool to Alert doctors to Right Treatment). Consensus validation. *International Journal of Clinical Pharmacology &* *Therapeutics*. 2008;46:72–83. 10.5414/cpp46072 18218287

[31] Lam MP, Cheung BM. The use of STOPP/START criteria as a screening tool for assessing the appropriateness of medications in the elderly population. *Expert review of clinical pharmacology*. 2012;5(2):187–97. 10.1586/ecp.12.6 22390561

[32] O'Mahony D, O'Sullivan D, Byrne S, O'Connor MN, Ryan C, Gallagher P. STOPP/START criteria for potentially inappropriate prescribing in older people: version 2. *Age and ageing*. 2015; 44(2):213–218. 10.1093/ageing/afu145 25324330 PMC4339726

[33] Ryan C, O'mahony D, Byrne S. Application of STOPP and START criteria: interrater reliability among pharmacists. *Annals of Pharmacotherapy*. 2009;43(7–8):1239–44. 19584381 10.1345/aph.1M157

[34] Stausberg J, Hasford J. Identification of adverse drug events: the use of ICD-10 coded diagnoses in routine hospital data. *Deutsches Arzteblatt International*. 2010;107(3):23 10.3238/arztebl.2010.0023 20140170 PMC2816787

[35] Coupland CA, Dhiman P, Barton G, Morriss R, Arthur A, Sach T, Hippisley-Cox J. A study of the safety and harms of antidepressant drugs for older people: a cohort study using a large primary care database. InNIHR Health Technology Assessment programme: Executive Summaries 2011. NIHR Journals Library. 10.3310/hta15280 21810375

[36] Lindquist M, Edwards IR. The WHO Programme for International Drug Monitoring, its database, and the technical support of the Uppsala Monitoring Center. *The Journal of rheumatology*. 2001;28(5):1180–1187. 11361210

[37] Hallas J, Harvald B, Gram LF. Drug related hospital admissions: the role of definitions and intensity of data collection, and the possibility of prevention. *Journal of internal medicine*. 1990;228(2):83–90. 2394974 10.1111/j.1365-2796.1990.tb00199.x

[38] Katzung BG, ed. Basic and clinical pharmacology 11th ed. New York: McGraw-Hill Education; 2010.

[39] Parfitt K, Martindale W. *Martindale*: *The complete drug reference* 36^th^ ed. London, UK: Pharmaceutical Press; 2009.

[40] Gallagher P, O'Mahony D. STOPP (Screening Tool of Older Persons' potentially inappropriate Prescriptions): application to acutely ill elderly patients and comparison with Beers' criteria. *Age and Ageing*. 2008;37(6):673–679. 10.1093/ageing/afn197 18829684

[41] Petty DR, Zermansky AG, Raynor DK. Evidence shows medication reviews by pharmacists point way forward. *Pharmaceutical journal*. 2001;267(7178):863–5.

[42] Silva RD, Macêdo LA, dos Santos Júnior GA, Aguiar PM, de Lyra Júnior DP. Pharmacist-participated medication review in different practice settings: Service or intervention? An overview of systematic reviews. *PloS One*. 2019;14(1):e0210312 10.1371/journal.pone.0210312 30629654 PMC6328162

[43] Lao CK, Ho SC, Chan KK, Tou CF, Tong HHY, Chan A. Potentially inappropriate prescribing and drug–drug interactions among elderly Chinese nursing home residents in Macao. *International Journal of Clinical Pharmacy*. 2013; 35(5): 805–812. 10.1007/s11096-013-9811-y 23812679

[44] Liu C-L, Peng L-N, Chen Y-T, Lin M-H, Liu L-K, Chen L-K. Potentially inappropriate prescribing (IP) for elderly medical inpatients in Taiwan: A hospital-based study. *Archives of Gerontology and Geriatrics*. 2012; 55(1):148–151. 10.1016/j.archger.2011.07.001 21820189

[45] Nagendra Vishwas H, Harugeri A, Parthasarathi G, Ramesh M. Potentially inappropriate medication use in Indian elderly: Comparison of Beers' criteria and Screening Tool of Older Persons' potentially inappropriate Prescriptions. *Geriatrics & Gerontology International*. 2012;12(3):506–14. 22239067 10.1111/j.1447-0594.2011.00806.x

[46] Gallagher P, Lang PO, Cherubini A, Topinková E, Cruz-Jentoft A, Errasquín BM, Mádlová P, Gasperini B, Baeyens H, Baeyens JP, Michel JP. Prevalence of potentially inappropriate prescribing in an acutely ill population of older patients admitted to six European hospitals. *European Journal of Clinical Pharmacology*. 2011;67(11):1175 10.1007/s00228-011-1061-0 21584788

[47] Hamilton H, Gallagher P, Ryan C, Byrne S, O'Mahony D. Potentially inappropriate medications defined by STOPP criteria and the risk of adverse drug events in older hospitalized patients. *Archives of internal medicine*. 2011; 171(11):1013–1019. 10.1001/archinternmed.2011.215 21670370

[48] Barry PJ, Gallagher P, Ryan C, O'Mahony D. START (screening tool to alert doctors to the right treatment)—an evidence-based screening tool to detect prescribing omissions in elderly patients. *Age and Ageing*. 2007; 36(6):632–638. 10.1093/ageing/afm118 17881418

[49] Chang CB, Yang SY, Lai HY, Wu RS, Liu HC, Hsu HY, Hwang SJ, Chan DC. Using published criteria to develop a list of potentially inappropriate medications for elderly patients in Taiwan. *Pharmacoepidemiology and drug safety*. 2012;21(12):1269–79. 10.1002/pds.3274 22517563

[50] Winit-Watjana W, Sakulrat P, Kespichayawattana J. Criteria for high-risk medication use in Thai older patients. *Archives of Gerontology and Geriatrics*. 2008;47(1):35–51. 10.1016/j.archger.2007.06.006 17675177

[51] Kim DS, Heo SI, Lee SH. Development of a list of potentially inappropriate drugs for the korean elderly using the delphi method. *Healthcare Informatics Research*. 2010;16(4):231–52. 10.4258/hir.2010.16.4.231 21818443 PMC3092136

[52] Thevelin S, El Mounaouar L, Marien S, Boland B, Henrard S, Dalleur O. Potentially Inappropriate Prescribing and Related Hospital Admissions in Geriatric Patients: A Comparative Analysis between the STOPP and START Criteria Versions 1 and 2. *Drugs & Aging*. 2019; 29:1–7. 10.1007/s40266-018-00635-8 30694444

[53] Lönnbro J, Wallerstedt SM. Clinical relevance of the STOPP/START criteria in hip fracture patients. *European Journal of Clinical Pharmacology*. 2017;73(4):499–505. 10.1007/s00228-016-2188-9 28050623 PMC5350233

[54] Karani MV, Haddad Y, Lee R. The role of pharmacists in preventing falls among America’s older adults. Frontiers in public health. 2016; 9;4:250. 10.3389/fpubh.2016.00250 PMC5101193 27882314

[55] YH Stevens GA, National Rao M., regional, and global trends in fasting plasma glucose and diabetes prevalence since 1980: systematic analysis of health examination surveys and epidemiological studies with 370 country-years and 2.7 million participants. The Lancet. 2011 7 2;378(9785):31. 10.1016/S0140-6736(11)60679-X 21705069

[56] Almeida TA, Reis EA, Pinto IV, Ceccato MD, Silveira MR, Lima MG, Reis AM. Factors associated with the use of potentially inappropriate medications by older adults in primary health care: an analysis comparing AGS Beers, EU (7)-PIM List, and Brazilian Consensus PIM criteria. *Research in Social and Administrative Pharmacy*. 2018 6 15. 10.1016/j.sapharm.2018.06.002 29934277

[57] Anathhanam S, Powis RA, Cracknell AL, Robson J. Impact of prescribed medications on patient safety in older people. *Therapeutic Advances in Drug Safety*. 2012; 3(4):165–74. 10.1177/2042098612443848 25083234 PMC4110850

[58] Weng MC, Tsai CF, Sheu KL. The impact of number of drugs prescribed on the risk of potentially inappropriate medication among outpatient older adults with chronic diseases. *QJM*: *An International Journal of Medicine*. 2013; 106(11): 1009–1015. 10.1093/qjmed/hct141 23836694

[59] Sheikh A, Rudan I, Cresswell K, Dhingra-Kumar N, Tan ML, Häkkinen ML, Donaldson L. Agreeing on global research priorities for medication safety: an international prioritisation exercise. *Journal of Global Health*. 2019;9(1). 10.7189/jogh.09.010422 PMC6393844 30842883

[60] Kuijpers MA, Van Marum RJ, Egberts AC, Jansen PA, OLDY (OLd people Drugs & dYsregulations) Study Group. Relationship between polypharmacy and underprescribing. *British Journal of Clinical Pharmacology*. 2008;65(1):130–3. 10.1111/j.1365-2125.2007.02961.x 17578478 PMC2291281

[61] Steinman MA, Seth Landefeld C, Rosenthal GE, Berthenthal D, Sen S, Kaboli PJ. Polypharmacy and prescribing quality in older people. *Journal of the American Geriatrics* Society. 2006;54(10):1516–23. 10.1111/j.1532-5415.2006.00889.x 17038068

[62] Henschel F, Redaelli M, Siegel M, Stock S. Correlation of incident potentially inappropriate medication prescriptions and hospitalization: an analysis based on the PRISCUS list. *Drugs-Real World Outcomes*. 2015;2(3):249–59. 10.1007/s40801-015-0035-4 27747571 PMC4883217

[63] Gates PJ, Meyerson SA, Baysari MT, Westbrook JI. The prevalence of dose errors among paediatric patients in hospital wards with and without health information technology: A systematic review and meta-analysis. *Drug safety*. 2019;42(1):13–25. 10.1007/s40264-018-0715-6 30117051

[64] Thevelin S, Spinewine A, Beuscart JB, Boland B, Marien S, Vaillant F, Wilting I, Vondeling A, Floriani C, Schneider C, Donzé J. Development of a standardized chart review method to identify drug‐related hospital admissions in older people. *British journal of clinical pharmacology*. 2018;84(11):2600–14. 10.1111/bcp.13716 30007041 PMC6177723

